# A Cycle-Based Data Aggregation Scheme for Grid-Based Wireless Sensor Networks

**DOI:** 10.3390/s140508447

**Published:** 2014-05-13

**Authors:** Yung-Kuei Chiang, Neng-Chung Wang, Chih-Hung Hsieh

**Affiliations:** Department of Computer Science and Information Engineering, National United University, Miaoli 36003, Taiwan; E-Mails: ykchiang@nuu.edu.tw (Y.-K.C.); x06231@gmail.com (C.-H.H.)

**Keywords:** base station, cell head, cycle leader, grid-based, wireless sensor networks

## Abstract

In a wireless sensor network (WSN), a great number of sensor nodes are deployed to gather sensed data. These sensor nodes are typically powered by batteries so their energy is restricted. Sensor nodes mainly consume energy in data transmission, especially over a long distance. Since the location of the base station (BS) is remote, the energy consumed by each node to directly transmit its data to the BS is considerable and the node will die very soon. A well-designed routing protocol is thus essential to reduce the energy consumption. In this paper, we propose a Cycle-Based Data Aggregation Scheme (CBDAS) for grid-based WSNs. In CBDAS, the whole sensor field is divided into a grid of cells, each with a head. We prolong the network lifetime by linking all cell heads together to form a cyclic chain so that the gathered data can move in two directions. For data gathering in each round, the gathered data moves from node to node along the chain, getting aggregated. Finally, a designated cell head, the cycle leader, directly transmits to the BS. CBDAS performs data aggregation at every cell head so as to substantially reduce the amount of data that must be transmitted to the BS. Only cell heads need disseminate data so that the number of data transmissions is greatly diminished. Sensor nodes of each cell take turns as the cell head, and all cell heads on the cyclic chain also take turns being cycle leader. The energy depletion is evenly distributed so that the nodes' lifetime is extended. As a result, the lifetime of the whole sensor network is extended. Simulation results show that CBDAS outperforms protocols like Direct, PEGASIS, and PBDAS.

## 1. Introduction

Rapid advances in sensor technology, wireless communications, and digital electronics have made it possible to develop low-cost, low-power and multi-functional small sensor nodes, which are capable of not only gathering and processing but of transmitting data as well [1–4]. A wireless sensor network (WSN) comprises a great number of small sensor nodes generally deployed over a large harsh field. We further web them together via wireless communication to gather data. WSNs can be employed in many applications such as environmental and habitat monitoring, target tracking, object detection, and intelligent transport monitoring [5–7].

This paper mainly addresses the problem of data transmission from all sensor nodes to a remote base station (BS), where the end-user can access the data. In a WSN, sensor nodes are generally deployed over a severe environment. In addition, these sensor nodes are typically powered by batteries so their energy is restricted. It is usually inconvenient or impossible to recharge or replace the batteries of the senor nodes in such a severe environment. Thus the network should be designed to endure. The energy consumption of sensor nodes is chiefly used in data transmission, especially over long distances. Since the location of the BS is remote, the energy consumed by each node to directly transmit its data to the BS is considerable and the node will die very soon. Therefore, it is important to use as few transmissions as possible to the BS and reduce the amount of data that needs to be transmitted to the BS [8].

Lots of improved approaches have been proposed. Most of these efforts focus on energy efficiency [8–13]. In order to diminish the data transmissions, only a few nodes are responsible for disseminating the data to the BS instead. On the other hand, the dissemination nodes aggregate their own data with the data sensed by others and then transmit it out to reduce the amount of data transmitted to the BS. Aggregated data moves from node to node, and finally a designated node transmits to the BS. It is our design goal to operate sensor nodes in an energy efficient manner to maximize the nodes' lifetimes, so as to further extend the lifetime of the whole network.

In this paper, we present a Cycle-Based Data Aggregation Scheme (CBDAS) for grid-based WSNs. In order to extend the lifetime of a WSN, we initially construct a grid-based infrastructure by partitioning the entire sensor field into a 2-D grid of cells. Each cell has a head (henceforth called a cell head) which is the node with the most residual energy, and the ordinary nodes which are the other nodes of the cell. In this paper, the cell head is equivalent to a cluster head, as in some cluster-based approaches. Each cell head is further linked together to form a cyclic chain. In each round, one cell head with the most residual energy on the chain takes a turn as cycle leader, responsible for directly transmitting data to the BS. In CBDAS, all ordinary nodes periodically transmit the sensed data to its cell head. After receiving the data from the ordinary nodes of the cell, the cell head aggregates it with its own data and then sends it out. Only cell heads need to disseminate data to the cycle leader. The ordinary sensor nodes just go into sleep mode based on the GAF protocol [14].

The rest of this paper is organized as follows: Section 2 briefly reviews some related works. In Section 3, our proposed scheme is described. The simulation results are discussed in Section 4. Finally, Section 5 draws the conclusions.

## 2. Related Work

Many WSN protocols have been developed in recent years to extend the system lifetime. Among of those, we briefly review some relevant designs: chain-based, cluster-based, grid-based, and diffusion-based.

In the chain-based protocol, PEGASIS [8] links all sensor nodes with a greedy algorithm to form a chain. Each node receives from one neighbor data fused with its own and then transmits to the other on the chain. Gathered data moves from node to node along the chain and finally a designated node called leader transmits to the BS. To reduce the average energy spent by each node per round, nodes take turns transmitting to the BS. EECB [11] improves PEGASIS by applying a distance threshold to avoid the formation of Long Links (LLs) on the chain to further distribute the energy load evenly among the nodes. Besides, EECB selects the node with minimum cost as leader to finally transmit gathered data to the BS. The cost is a function of the remaining energy of the node over the distance between the node and the BS. ECBSN [15] adopts 2-layer hierarchical chains. In the low layer, ECBSN based on PEGASIS adopts multiple chains instead. Each chain has a leader, the node with the most remaining energy. ECBSN further links the leader of each low layer chain to form a single high layer chain. In the high layer chain, the node with the shortest distance to the BS is selected as the high layer leader, responsible for transmitting the aggregated data finally to the BS. In the data transmission phase, the token passing mechanism of each low layer chain is the same as that of PEGASIS. After receiving the gathered data, the leader of each low layer chain transmits to the high layer leader which eventually transmits to the BS.

In the cluster-based protocol, LEACH [16] combines the ideas of cluster-based routing and media access together with application-specific data aggregation for WSNs. In LEACH, distributed clusters are self-organized by the nodes into a local cluster. Each cluster has a head responsible for aggregating data and transmitting it to the remote BS. LEACH incorporates randomized rotation of cluster head position to evenly distribute the energy load among all the nodes so as to prolong the system lifetime. In GROUP [17], one of sinks proactively, dynamically and randomly constructs a cluster grid structure to relay queries and data packets. Only a small part of the sensor nodes will participate in the election of cluster heads. Cluster heads can expediently aggregate data to reduce the volume of data packets. GROUP can also distribute the energy load among the sensor nodes in the network. Vidhya and Dananjayan's model [4] modifies the LEACH protocol and allows cluster heads to form a multi-hop backbone by incorporating a cooperative MIMO scheme [9] through the selection of cooperative sending and receiving nodes.

In the grid-based protocol, TTDD [18] builds the grid on a per-source basis. A source transmits data by building a grid structure. Each grid point has a dissemination node responsible for storing and forwarding data. A sink sends the immediate dissemination node a query which is in turn forwarded towards the source. The query forwarding process lays the information of the path to the sink to enable the requested data from the source to the sink. Unlike TTDD's per source-based grid structure, CODE [19] divides the whole sensor field into grids. Each grid has one coordinator which acts as an intermediate node to cache and relay data. In data announcement and query transfer phases, CODE establishes a data dissemination path first so that the source can send data to the mobile sink along the path. EEDD [10] also divides the whole sensor field into small virtual grids so that each grid has a head to forward data. Each grid is further divided into four sub-grids if an event is unceasing. Working nodes in the sub-grids are scheduled to stay active according to their corresponding sub-grid time slot. When a target is detected and needs to be sent to the sink, the data packets are forwarded by the working node to the neighboring grid head toward the sink. With an adaptive scheduling scheme, EEDD can support both target and sink mobility. PBDAS [20] is for grid-based WSNs with a single chain, simply formed by repeatedly linking the cell heads from the farthest row left to right then the next farthest row right to left until the nearest row of the BS. In PBDAS, choosing a cell head according to the energy level increases the lifetime and robustness of the WSN.

In the diffusion-based protocol, Directed Diffusion [21] proposes a data centric dissemination protocol for sensor networks. Data generated by sensor nodes is named using attribute-value pairs so that a node requests data just by sending interest messages for named data. Data matching the interests is then drawn down toward that node. Intermediate nodes can cache and aggregate data. Directed Diffusion applies the technique of initial low-rate data flooding to establish a reinforced path from source to destination. Thus, data packets are then forwarded through that route instead of being broadcast. EADD [22] changes the node's forwarding moment that depends on each node's available energy, allowing the nodes with higher available energy to response more quickly than those with lower available energy. EADD solves uneven energy consumption problem to achieve balanced nodes' energy distribution and extend the network lifetime. Wan *et al.* [23] proposed a gradient model based on one-phase Directed Diffusion. This model evenly distributes the energy load among all the nodes in the network. It also takes into account the hop count from the sink to an intermediate node to satisfy some real-time applications.

## 3. The Proposed Scheme

In this section, we will describe the proposed protocol for WSNs in detail. The proposed scheme has four primary phases: grid construction, cell head election, cycle formation, and data transmission.

### 3.1. Grid Construction

In CBDAS, the whole sensor field is partitioned into a logical grid of *M* × *N* cells, where *N* is even. Each cell has an ID, containing sensor nodes. A sensor node can calculate its cell ID [*C_X_*, *C_Y_*] from its geographic location (*x*, *y*) as follows:

(1) $$C_{X}=\left\lfloor \frac{x-x_{0}}{\alpha }\right\rfloor ,\;C_{Y}=\left\lfloor \frac{y-y_{0}}{\alpha }\right\rfloor ,$$

where (*x*_0_, *y*_0_) is the location of the virtual origin set at the network initialization stage, *α* is the cell size, and ⌊*k*⌋ is the largest integer not greater than *k*. For simplicity, we assume that all cell IDs are positive.

**Figure 1.**
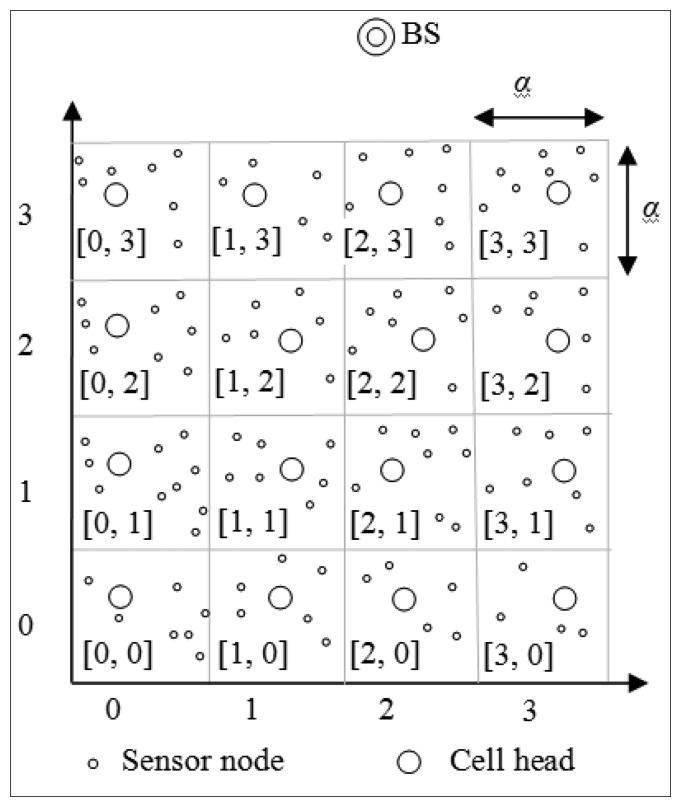
The logical grid structure.

Figure 1 illustrates an example sensor field partitioned into a logical grid of 4 × 4 cells, where the cell IDs are enclosed by square brackets. From left to right, the cell IDs are [0, 0], [1, 0], [2, 0] and [3, 0] in the first row, the cell IDs are [0, 1], [1, 1], [2, 1] and [3, 1] in the second row, and so on and so forth.

In CBDAS, each cell has a head and ordinary nodes, the other nodes of the cell. The cell head is responsible for aggregating data transmitted from ordinary nodes in the same cell and in turn transmitting it to the neighboring cell head toward the cycle leader.

### 3.2. Cell Head Election

In CBDAS, each node maintains a table to keep its own geographic location, cell ID, and the current cell head. Initially, each sensor node computes itself which cell it belongs to using Equation (1). Each sensor node *s* will then compete for being a cell head by broadcasting a *Head_electing* message with its location after waiting for a certain period of time *t*(*s*), which is determined as follows:

(2) t(s)=K/energy(s)

where *K* is a constant set at network initialization stage and *energy*(*s*) is the residual energy of node *s*. Hence, a node with more residual energy has a higher probability of becoming a cell head. Once a node in the same cell receives *Head_electing*, it will give up its attempt since its residual energy is less. When there are multiple announcements, the node with the smallest location will win the competition. Location *L*_1_(*x*_1_, *y*_1_) is smaller than *L*_2_(*x*_2_, y_2_) if *x*_1_ is smaller than *x*_2_ or both *x*_1_ and *x*_2_ are equal but *y*_1_ is smaller than *y*_2_. Subsequently, the cell head will broadcast a *Head_confirming* message with its location to announce its leadership. The ordinary nodes update their own table with the location of the new cell head. As a result, there is one cell head in each cell after election. Since a cell head node consumes much more energy than an ordinary node, our goal is to try to evenly distribute the energy load among the nodes in the network so that no overly used nodes will exhaust energy before the others.

### 3.3. Cycle Formation

**Figure 2.**
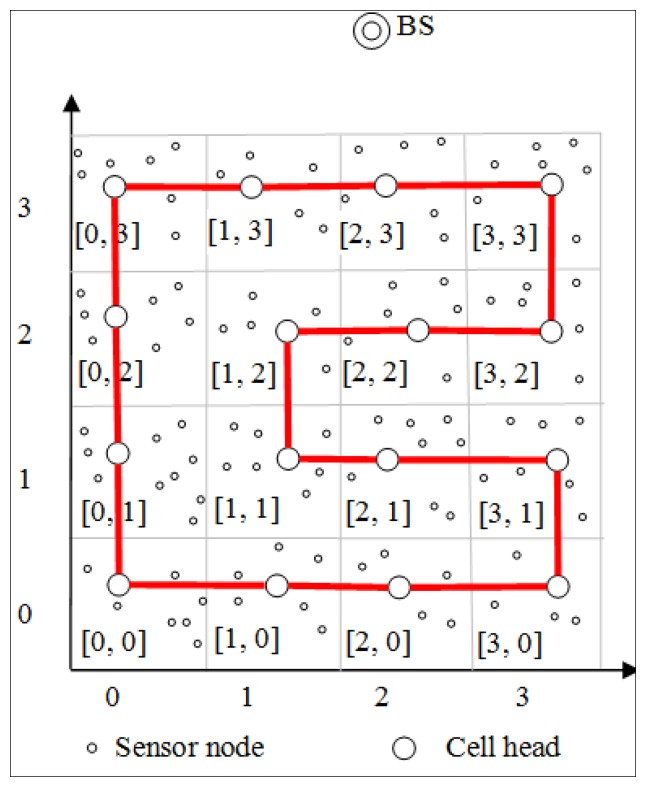
A cyclic chain.

The main idea in CBDAS is to construct a cyclic chain by linking all cell heads so that sensed data can be disseminated in two directions along the chain to the BS. Once the cell head of each cell is determined for the first round, it starts finding its downlink cell head clockwise in the neighboring cell by using Algorithm 1. After determining its downlink, the cell head with cell ID [*C_x_*, *C_y_*] sends a *Cycle_forming* packet containing its own cell ID to the downlink. After receiving the packet, the downlink cell head saves the information and replies with a *Cycle_reply* packet containing its own cell ID to the uplink cell head from which it receives the *Cycle_forming* packet. Likewise, the uplink cell head saves the information in the *Cycle_reply* packet. In this way, the cell head in each cell caches the information of its uplink and downlink. The cell heads of the whole network are finally linked together to form a Hamiltonian cycle, as shown in Figure 2. As a result, the data can be transmitted both clockwise and counterclockwise simultaneously in CBDAS.


**Algorithm 1** FindDownlinkCell (CELL)
{ δx = δy = 0; **If** (CELL.Cx == 0)/* The cells of the leftmost column */  **If** (CELL.Cy == N−1) δx = 1;/* The cell of left top */  **Else** δy = 1; **Else If** (CELL.Cx == M−1)/* The cells of the rightmost column */  **If** (CELL.Cy % 2 == 0) δx = −1;  **Else** δy = −1; **Else If** (CELL.Cx == 1) δy = −1;/* The cells of the second column */ DOWNLINKCELL.Cx = CELL.Cx + δx; DOWNLINKCELL.Cy = CELL.Cy + δy; **return** DOWNLINKCELL;}


### 3.4. Data Transmission

For gathering data in each round, the BS chooses the cell head with the most residual energy as the cycle leader, responsible for receiving data from its two neighboring cell heads, aggregating with its own data, and eventually transmitting the aggregated data to the BS. In each round, when the cycle leader receives the request from the BS, it will send two tokens *t*_1_ and *t*_2_ to its two neighboring cell heads, respectively. Token *t*_1_ will be passed recursively along the cycle clockwise to the next cell head. Conversely, token *t*_2_ will be passed recursively along the cycle counterclockwise to the next cell head. Both tokens travel along the cyclic chain in opposite directions. Once a cell head receives the second token from the other direction, it drops the token and cuts the cycle, making itself as one end and the sender of the second token as the other end. As a result, both end cell heads respectively transmit their own aggregated data to the cycle leader in the opposite direction.

**Figure 3.**
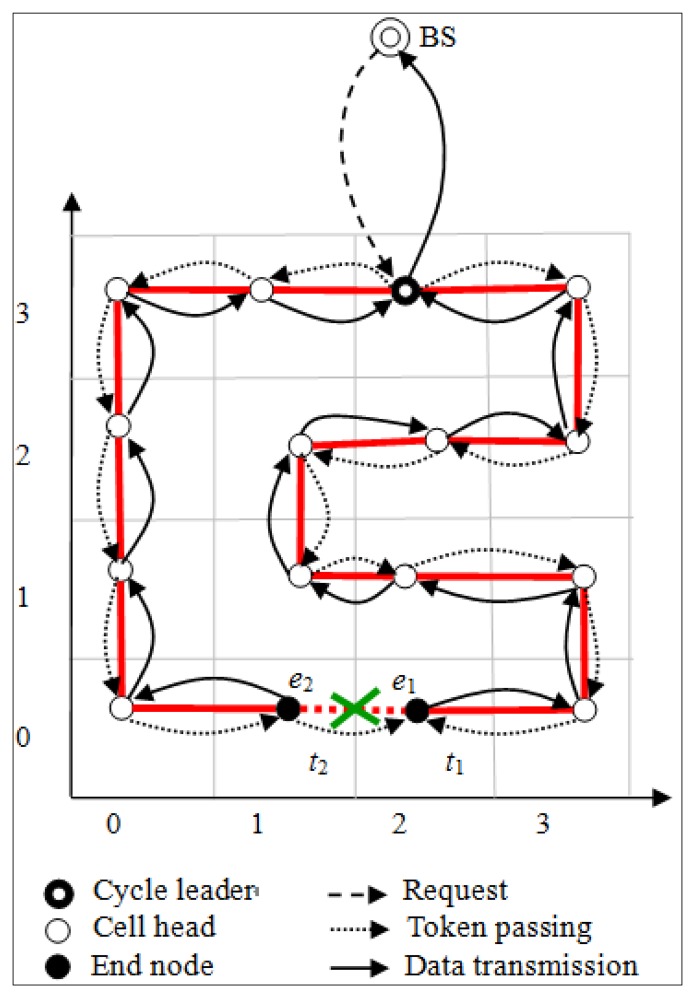
Scheme for data gathering in each round.

For example, as shown in Figure 3, when cell head *e*_1_ receives the second token *t*_2_ passed along the cycle counterclockwise, it drops *t*_2_ and cuts the cycle, making itself as one end and *e*2 as the other end. Likewise, when cell head *e*_2_ receives the second token *t*_1_ passed along the cycle clockwise, it drops *t*_1_ and cuts the cycle, making itself as one end and *e*1 as the other end. Accordingly, both *e*_1_ and *e*_2_ respectively transmit their own aggregated data to the cycle leader in the opposite direction.

### 3.5. Cycle Maintenance

Each cell head on the cyclic chain plays an important role in transmitting data. The data transmission path is actually maintained by each cell head. As long as all cell heads remain active, the data transmission path will work normally. When the residual energy of a cell head is below a threshold *E_head_*, it will broadcast a request to reelect a new cell head. All ordinary nodes with the residual energy above *E_head_* will take part in the competition utilizing the cell head election procedure described above.

**Figure 4.**
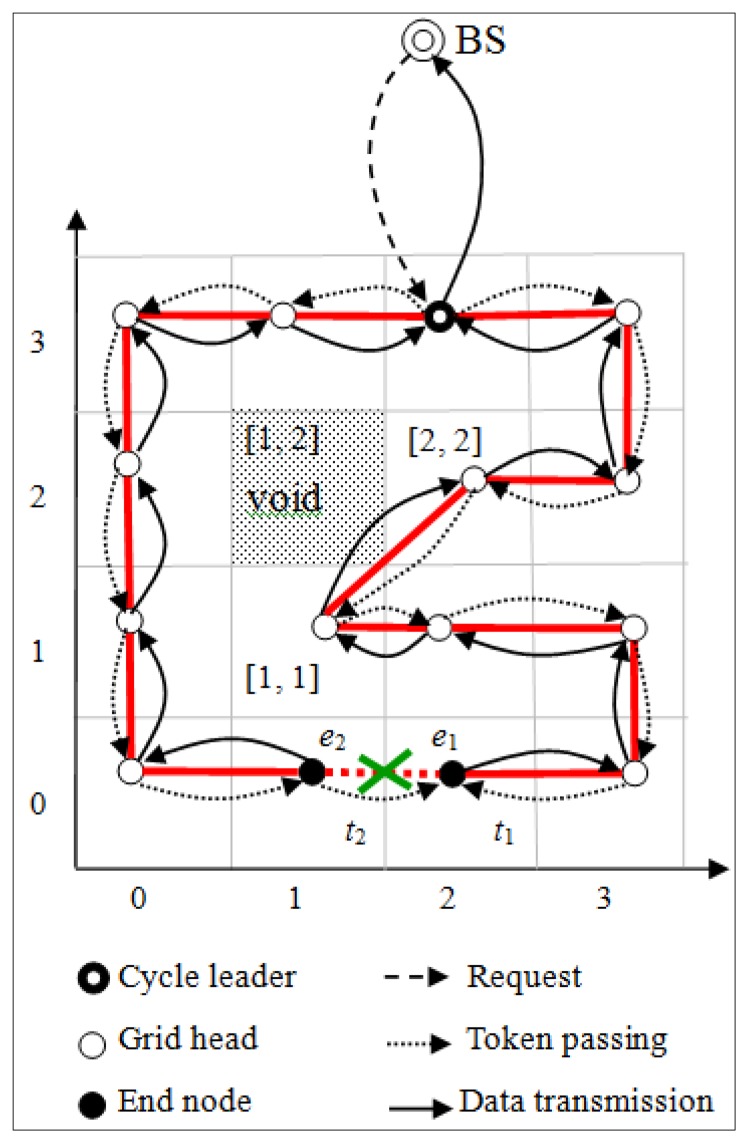
A void cell.

It is possible for a cell head to transmit data to a void cell, where all nodes are dead. A void cell is detected by a timeout. When a cell head never receives acknowledgement *Ack_cycle* from the uplink cell head for a certain time, it broadcasts a *Uplink_poll* message containing the IDs [*X_m_*, *Y_m_*] and [*X_d_*, *Y_d_*] of the uplink cell and itself. The cell head with downlink the same as [*X_m_*, *Y_m_*] will set its downlink to be [*X_d_*, *Y_d_*] and reply a *Uplink_reply* message containing its own ID [*X_u_*, *Y_u_*]. After receiving *Uplink_reply*, the cell head with ID [*X_d_*, *Y_d_*] will set its uplink to be [*X_u_*, *Y_u_*] and transmit the aggregated data to the cell head of the new uplink cell. For example, consider the cell [1, 2] is a void cell, where all nodes are dead, as shown in Figure 4. When the cell head of cell [1, 1] never receives acknowledgement *Ack_cycle* from the uplink cell [1, 2] for a certain time, it broadcasts a *Uplink_poll* message with the IDs [1, 2] and [1, 1] of the uplink cell and itself. Owing to the cell head of cell [2, 2] with downlink the same as [1, 2], it will set the downlink to be [1, 1] and reply a *Uplink_reply* message containing its own ID [2, 2]. After receiving *Uplink_reply*, the cell head with ID [1, 1] will set its uplink to be [2, 2] and transmit the aggregated data to the cell head of the new uplink cell [2, 2].

### 3.6. Discussion

In order to concentrate our attention on designing an energy efficient routing protocol, we made some assumptions:

1. All sensor nodes are stationary after deployment.
2. Each sensor node is aware of its own geographic location based on GPS or some other technique [24,25].
3. Each senor node is also aware of its residual energy.
4. The sensor nodes are homogeneous and wireless channels are bidirectional.

Like most of the applications in WSNs, all sensor nodes are stationary after deployment in CBDAS. As for the second assumption, being location aware, each sensor node can determine to which cell it belongs, so that sensor nodes can be clustered into cells. As a result, the grid infrastructure is constructed in the field. Besides, sensor nodes are energy aware so they can compete for the election of the cell head in their own cell. For simplicity, the sensor nodes are homogeneous, and wireless channels are bidirectional in terms of general definition.

The scenario of our application is that each sensor node equipped with GPS is stationary after deployment. Initially, each sensor node determines which cell it is located in using Equation (1), and as a result the grid infrastructure is constructed. Each sensor node is aware of its residual energy to compete for being the head of its cell. So is the cell head to be designated as the cycle leader. Our clustering approach is static (not dynamic) so the GPS can be turned off after the grid infrastructure is constructed. Sadly, a sensor node is useless if its geographic location is not available, even though the other functions are normal.

Some issues of the assumptions are discussed in the related literature [18,19,26–28]. In [26,27], the authors proposed geographic routing strategies using the partial position information. In [18,19], the authors proposed data dissemination schemes with mobile sinks. In [28], Wu *et al.* proposed deployment and topology control schemes in heterogeneous WSNs. In spite of the fact the abovementioned issues are important, those issues are not our key issue. In this paper, we focus on a load balancing strategy to improve the chain-based data aggregation for WSNs.

We could have constructed a linear chain with two ends like other approaches [8,11,15]. However, we generate a cyclic chain instead. We use a simple token passing approach initiated by the cycle leader to start the data transmission. In a given round, the cycle leader passes each of both neighboring cell heads a token in the opposite direction. When both tokens meet, the cyclic chain will be cut with two ends having almost the same hops to the cycle leader. Both end nodes subsequently transmit the gathered data simultaneously so that the data transmission will be more efficient. This benefits real time data transmission a lot.

As a chain constructed by using the greedy algorithm, the neighbor distances will increase gradually. Besides, the gathered data will get bigger and bigger after aggregating. According to the first order radio model [5], as stated later in Section 4, the transmission consumption for a bigger message over a longer distance will greatly increase. As a result, this causes a portion of the nodes in the network to dissipate energy so extremely that the network lifetime shortens. However, the neighbor distances are approximately equal on a cyclic chain. The aggregated data is divided into two portions transmitted along two opposite paths. The energy load is evenly distributed so that the network lifetime is extended.

In general, the chain-based routing (such as PEGASIS) suffers with its long distance and the cluster-based routing (such as LEACH) suffers the hot-spot effect for head (leader) nodes. The grid-based routing is a kind of the cluster-based routing. In this paper, we focused on the load balancing strategy to improve the chain-based data aggregation for WSNs. The aim of this paper is to provide another one solution for energy efficient WSNs. We construct an architecture that combines the concepts of the chain-based routing and grid-based routing. In addition, we add the idea of load balancing with the cycle-based approach for distributing data transmission to reduce energy consumption.

In CBDAS, the whole sensor field is partitioned into a logical grid of *M* × *N* cells, each has a head. All cell heads are further linked together to form a cyclic chain. The complexity of this algorithm is therefore *O*(*M* × *N*). As for other algorithms [8,11,15] using the greedy method, then the complexity is *O*(*n*^2^), where *n* is the number of sensor nodes and *n* > *M* × *N*.

## 4. Simulation Results

We use the first order radio model [5] to evaluate the energy consumption of each node. According to this model, a radio dissipates *E_elec_* = 50 nJoule/bit to run the transmitter or receiver circuitry. *E_elec_* is the energy consumption of the circuit itself. Assuming *d*^2^ energy loss, where *d* is the distance between nodes, a transmission amplifier at the sender node further consumes *E_amp_d*^2^, where *E_amp_* = 100 pJoule/bit/m^2^. *E_amp_* is the energy consumed by the amplifier when transmitting packets. Thus, to transmit a *k*-bit message a distance *d* using this radio model, the radio expends:

(3) $$E_{Tx}(k,d)=E_{\text{elec}}\times k+E_{\text{amp}}\times k\times d^{2}$$

and to receive this message, the radio expends:

(4) $$E_{Rx}(k)=E_{\text{elec}}\times k$$

Receiving a message is not a low cost operation using these parameter values. Protocols should thus try to minimize not only the transmission distances but also the numbers of transmission and reception operations for each message. We can generalize the total transmission consumption as follows:

(5) $$E_{\text{total}}(k)=(E_{\text{elec}}\times k+E_{\text{amp}}\times k\times d^{2})+(E_{\text{elec}}\times k)$$

In the following, we compare the performance of the proposed CBDAS with that of the Direct, PEGASIS [8], and PBDAS [20] protocols. The direct approach is simply for each node to transmit its data directly to the BS. In our simulations, the energy consumed for data aggregation is assumed to be 5 nJoule/bit/message. The simulation parameters are shown in Table 1.

To widely evaluate the impact of some factors, we changed the value of parameters in some cases. We applied an initial energy per node of 0.25 J, 0.5 J, and 1.0 J to evaluate the impact of initial energy. We applied a grid of 6 × 6, 8 × 8, and 10 × 10 cells to evaluate the impact of number of cells. We also applied 100, 200, 300, 400, 500, and 600 nodes to evaluate the impact of node density.

### 4.1. Impact of Initial Energy

**Figure 5.**
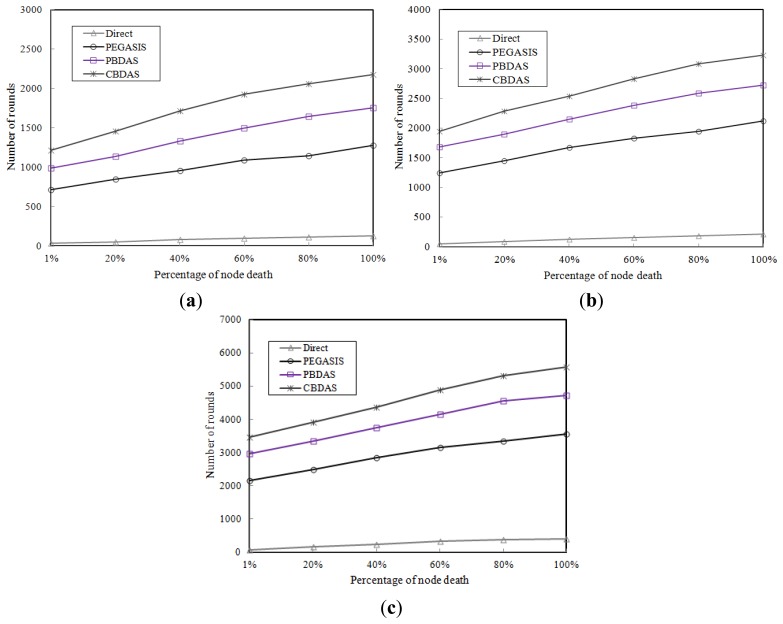
Number of rounds when 1%, 20%, 50%, and 100% nodes die. (**a**) Each node with initial energy 0.25 J; (**b**) Each node with initial energy 0.5 J; (**c**) Each node with initial energy 1.0 J.

To evaluate the impact of initial energy, we ran simulations to determine the number of rounds of data gathering when 1%, 20%, 50%, and 100% nodes die using Direct, PEGASIS, PBDAS, and CBDAS with each node having the same initial energy level. Figures 5a–c show the results of simulations with initial energy per node of 0.25 J, 0.5 J, and 1.0 J, respectively. In CBDAS, the sensor filed was partitioned into a grid of 10 × 10 cells. As the results show, CBDAS is better than the other approaches. On average, CBDAS has approximately 1.6 times more rounds compared to PEGASIS when 1%, 20%, 50%, and 100% nodes die. This is because the distances between nodes become longer and the nodes have to become leaders more frequently with a larger percentage of node deaths.

### 4.2. Impact of the Number of Cells

**Figure 6.**
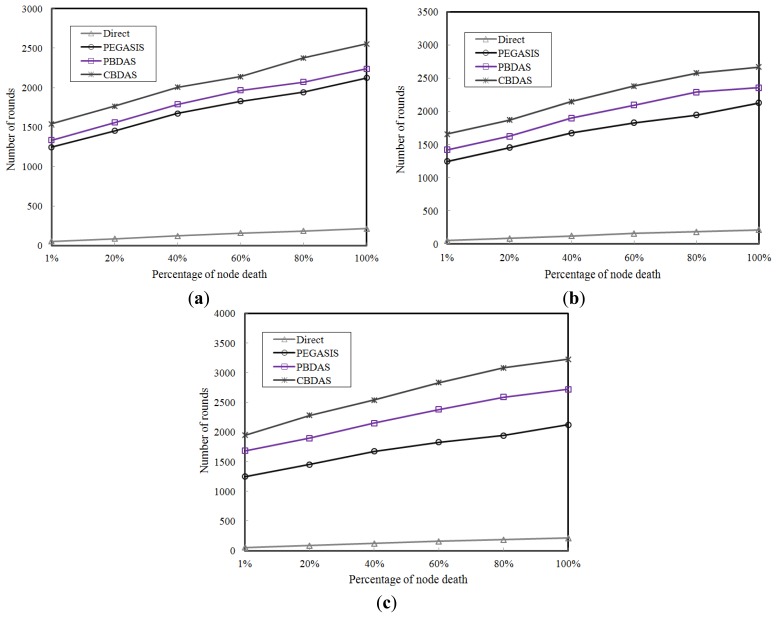
Impact of the number of cells. (**a**) A grid of 6 × 6 cells; (**b**) A grid of 8 × 8 cells; (**c**) A grid of 10 × 10 cells.

To compare the impact of the number of cells on CBDAS, we partitioned the sensor field into a grid of 6 × 6, 8 × 8, and 10 × 10 cells, respectively. The numbers of cells demonstrated in Figures 6a–c are 6 × 6, 8 × 8, and 10 × 10, respectively. As shown in these figures, CBDAS is better than the other approaches. For the number of rounds compared to PEGASIS, Figure 6a shows that CBDAS has approximately 1.2 times more for a grid of 6 × 6 cells, Figure 6b shows that CBDAS has approximately 1.3 times more for a grid of 8 × 8 cells, and Figure 6c shows that CBDAS has approximately 1.6 times more for a grid of 10 × 10 cells.

In this comparison, we realize that a grid of 10 × 10 cells is the most appropriate for CBDAS by applying the same parameters of 300 nodes used with initial energy of 0.5 J per node. According to Equation (5), the shorter the distance, the less energy the node consumes. In CBDAS, the ordinary nodes only need to transmit the data locally to their cell head located at a comparatively short distance. The energy consumed by them is thus reduced. Besides, the cell head also only transmits aggregated data to its uplink cell head on the chain within a shorter distance.

### 4.3. Impact of Node Density

**Figure 7.**
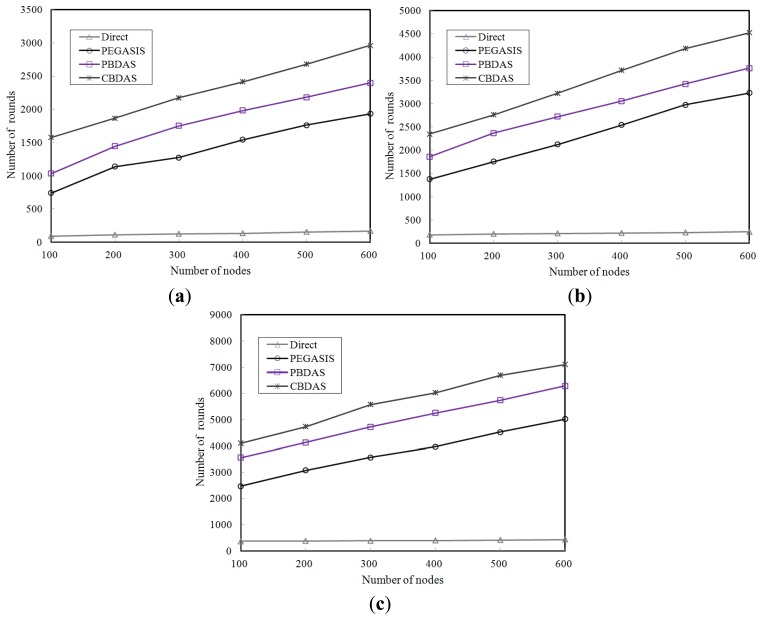
Impact of node density. (**a**) Each node with initial energy 0.25 J; (**b**) Each node with initial energy 0.5 J; (**c**) Each node with initial energy 1.0 J.

To study the impact of node density on each approach, we varied the number of nodes from 100 to 600 nodes with different initial energy. Figures 7a–c show the number of rounds of each approach at different node densities with initial energy 0.25 J, 0.5 J, and 1.0 J per node, respectively. The results show that the number of rounds of each approach increases significantly as node density multiplies except for the Direct protocol. CBDAS is also better than the other approaches as illustrated in the results. In CBDAS, as the node density multiplies, the quantity of nodes in each cell increases. The load of the cell head in each cell was thus shared among more nodes. As a result, the energy consumed by each node was not so significant that the number of rounds using CBDAS substantially increased. On average, CBDAS has approximately 1.6 times more rounds compared to PEGASIS, over the three different initial energy levels.

### 4.4. Network Lifetime

**Figure 8.**
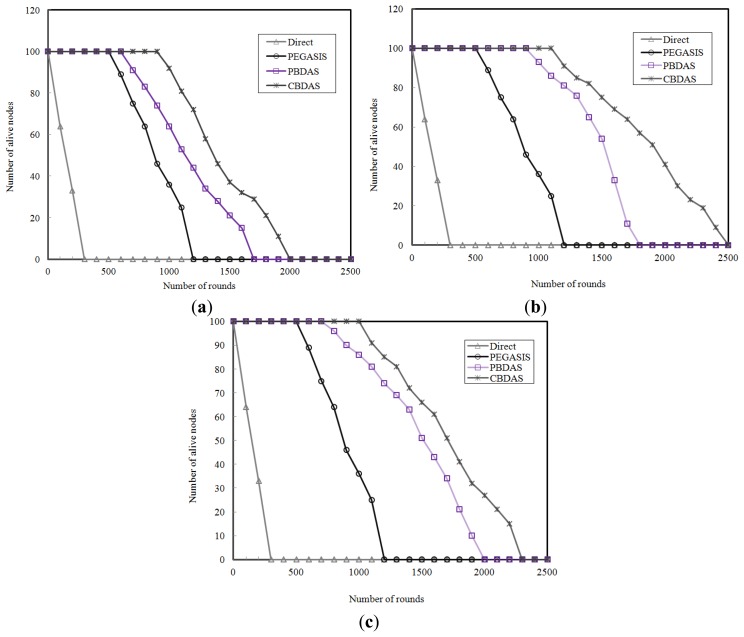
Number of alive nodes over time. (**a**) A grid of 6 × 6 cells; (**b**) A grid of 8 × 8 cells; (**c**) A grid of 10 × 10 cells.

We also study the network lifetime of each approach. A sensor node is considered to be dead if it doesn't have enough energy to receive or transmit data. We ran the simulations using the numbers of cells 6 × 6, 8 × 8, and 10 × 10, respectively, for CBDAS. As shown in Figures 8a–c, CBDAS has longer network lifetime than the other approaches. The simulation result shows that CBDAS lasts on average 1.3 times more the number of rounds than PEGASIS. It also shows that a grid of 10 × 10 cells is the most appropriate among the three numbers of cells for CBDAS. Figure 8c shows that the number of dead nodes is increasing after 2000 rounds for CBDAS but after 1200 rounds for PEGASIS though. In CBDAS, each node in a cell takes turn to be cell head and each cell head takes turn to be cycle leader, the energy depletion is evenly distributed so that the nodes' lifetime extend. As a result, the lifetime of the whole sensor network is prolonged.

### 4.5. The First Round Energy Consumed

Based on the assumption that all nodes start with an equal energy level, we compared the consumed energy of the first round of each approach rather than each round. We thus conducted a simulation to compare each approach the first round consumed energy in terms of the node density. We varied the number of nodes from 100 to 600 to evaluate the impact of node density for the first round.

**Figure 9.**
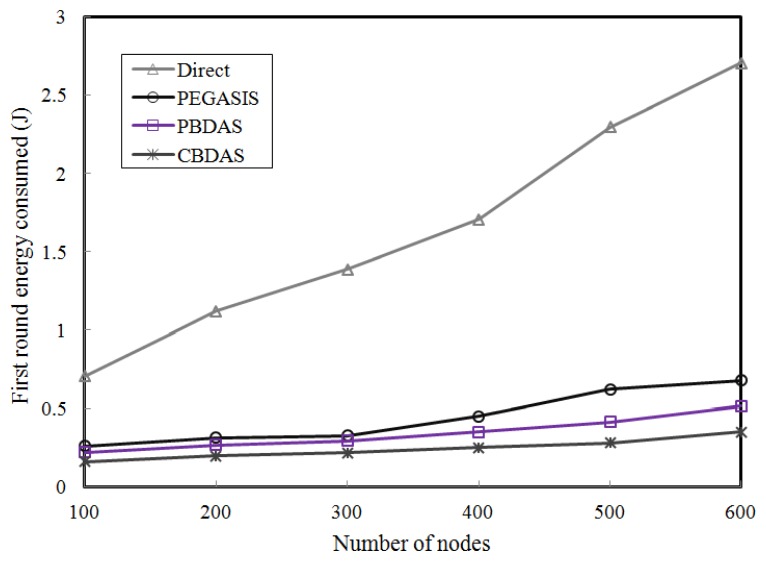
The first round energy consumed.

As shown in Figure 9, the first round consumed energy of CBDAS goes up quite slowly as the number of nodes increase. Unlike the other approaches, CBDAS builds the transmission path only once in the cycle formation phase. The overhead is only expended subsequently in maintaining the transmission path. The transmission path strategy is helpful to reduce the energy expenditure.

### 4.6. Total Consumed Energy

**Figure 10.**
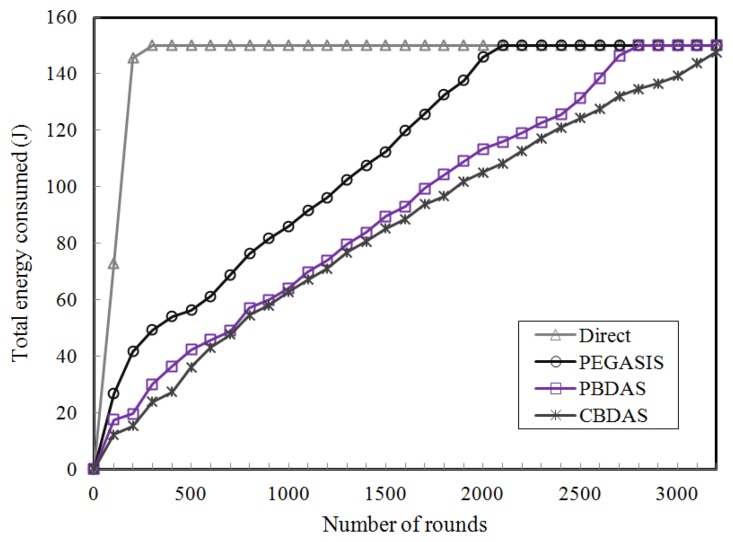
Total consumed energy over time.

The total consumed energy includes communication energy dissipation, computation energy dissipation, and sensing energy dissipation. In Figure 10, the total consumed energy is plotted against the number of data gathering rounds. As the number of rounds increases, the total consumed energy also increases. While the number of rounds increases, the total consumed energy of CBDAS increases the most steadily among those approaches. Since the energy consumption is evenly distributed among sensor nodes in CBDAS, the number of rounds can be over 3000. The total energy consumed increases abruptly when using Direct and PEGASIS though.

## 5. Conclusions

In this paper, we proposed a Cycle-Based Data Aggregation Scheme for grid-based WSNs. To achieve this goal, we first construct the grid infrastructure by partitioning the whole field into a grid of cells. Each node determines which cell it belongs to by a simple arithmetic operation. The formation of the cyclic chain is easy and the cost of its maintenance is also not high. To evenly distribute the energy depletion, the node with most residual energy in each cell is chosen to be the cell head. Only the cell head needs to forward the aggregated data of its own cell to the leader of cyclic chain. Also, the cell heads on the cyclic chain take turns to be cycle leader to transmit the aggregated data to the BS finally. As a result, the lifetime of the sensor nodes extends so as to prolong the lifetime of the whole sensor network. Choosing a cell head according to the energy level increases the lifetime and robustness of the WSN. Our simulations show that CBDAS outperforms the other approaches in terms of initial energy level, network size, and node density.

## Figures and Tables

**Table 1.** Parameters for the simulation.

**Parameters**		**Values**		
Network area		100 m × 100 m		
Location of BS		(50, 150)	
Initial energy		0.5 J/node	
Number of cells		10 × 10	
Number of sensor nodes		300	
Packet length		2,000 bits	
